# Modular glycosphere assays for high-throughput functional characterization of influenza viruses

**DOI:** 10.1186/1472-6750-13-34

**Published:** 2013-04-15

**Authors:** Sven N Hobbie, Karthik Viswanathan, Ido Bachelet, Udayanath Aich, Zachary Shriver, Vidya Subramanian, Rahul Raman, Ram Sasisekharan

**Affiliations:** 1 Singapore-MIT Alliance for Research and Technology, Singapore 138602, Singapore; 2 Harvard-MIT Division of Health Sciences and Technology, Koch Institute for Integrative Cancer Research, Department of Biological Engineering, Massachusetts Institute of Technology (MIT), Cambridge, MA 02139, USA

## Abstract

**Background:**

The ongoing global efforts to control influenza epidemics and pandemics require high-throughput technologies to detect, quantify, and functionally characterize viral isolates. The 2009 influenza pandemic as well as the recent in-vitro selection of highly transmissible H5N1 variants have only increased existing concerns about emerging influenza strains with significantly enhanced human-to-human transmissibility. High-affinity binding of the virus hemagglutinin to human receptor glycans is a highly sensitive and stringent indicator of host adaptation and virus transmissibility. The surveillance of receptor-binding characteristics can therefore provide a strong additional indicator for the relative hazard imposed by circulating and newly emerging influenza strains.

**Results:**

Streptavidin-coated microspheres were coated with selected biotinylated glycans to mimic either human or avian influenza host-cell receptors. Such glycospheres were used to selectively capture influenza virus of diverse subtypes from a variety of samples. Bound virus was then detected by fluorescently labelled antibodies and analyzed by quantitative flow cytometry. Recombinant hemagglutinin, inactivated virus, and influenza virions were captured and analyzed with regards to receptor specificity over a wide range of analyte concentration. High-throughput analyses of influenza virus produced dose–response curves that allow for functional assessment of relative receptor affinity and thus transmissibility.

**Conclusions:**

Modular glycosphere assays for high-throughput functional characterization of influenza viruses introduce an important tool to augment the surveillance of clinical and veterinarian influenza isolates with regards to receptor specificity, host adaptation, and virus transmissibility.

## Background

Influenza viruses are a significant cause of morbidity and mortality worldwide [1,2]. Besides the seasonal influenza epidemics caused by H1N1 and H3N2 influenza virus strains, new strains of influenza virus emerge periodically with pandemic potential. Despite the extensive network in place to monitor influenza virus evolution through mutation and recombination, public health laboratories still fail to detect novel strains of influenza and differentiate those that are primarily animal-adapted from those with true pandemic potential. For example, the outbreak of the swine-origin H1N1 pandemic in spring 2009 [3] hit the medical community unprepared, even though the initial transmission from swine to humans occurred months before, and prior to that it is believed to have circulated undetected in swine for years [4]. This underscored the gap in our ability to detect and characterize emerging strains before the widespread onset of disease in the population. Early detection of virus strains with pandemic potential is important, as early detection of an outbreak is critical to generate and stockpile sufficient quantities of vaccines and anti-virals to limit the spread of the disease.

One of the challenges in detecting emerging strains is that the factors leading to the generation of a pandemic virus are complex and poorly understood. At a functional level, however, it is clear that for a virus to have pandemic potential it must be capable of human-to-human aerosol transmission and there must exist a substantial population that is immunologically naïve to the strain of virus [5]. Poor human-to-human transmissibility of avian-adapted H5N1 strains causing “bird flu”, for example, seems to be the major impediment to more serious outbreaks [6,7]. Recent news on H5N1 variants capable of efficient aerosol transmission in ferrets, however, suggest that a few mutations may be sufficient to render bird flu highly transmissible in ferrets and possibly humans [8,9]. Therefore, the development of assays that identify subtypes and strains that have the potential to make the jump to humans from animal reservoirs is vitally important for disease surveillance and public health.

We have previously elucidated the role of the influenza hemagglutinin (HA) in aerosol transmissibility [7,10-17]. HA binding to cell surface glycans present on cells of the upper respiratory tract is the key initial step in viral infection; indeed, HA has been found to be one of the key viral genes involved in infectivity and transmission [11]. Together, these studies on seasonal and pandemic influenza strains have provided a comprehensive understanding of how high-affinity binding to the distinct topology of ‘long’ α2,6 linked sialylated glycans is a necessary step in efficient aerosol transmission (Additional file 1).

Current surveillance methods include genotyping of viral isolates using PCR to identify their type and subtype, as well as comparing the antigenicity of newly identified virus strains to existing strains. Despite comprehensive genotypic and phenotypic analyses, it is often difficult to functionally type the virus. Given the observed correlation between high affinity to long α2-6 sialylated glycans and efficient transmission, we reasoned that a surveillance strategy involving the typing of virus strains, and more specifically, viral HAs based on their affinity to long α2-6 sialylated glycan would provide a robust methodology to detect and predict the transmissibility and therefore pandemic potential of emerging strains.

Traditionally, receptor specificities of avian- and human-adapted influenza viruses are determined using a red-blood cell (RBC) agglutination assay. RBCs from species such as chicken, turkey, horses, guinea pigs and humans have been used in such assays [18,19]. RBCs have also been used in conjunction with sialidases and sialyltransferase to present certain glycan structures, for example exclusively containing either α2-3 or α2-6 linked sialic acid [20,21]. This type of assay however is inherently limited in that it fails to account for receptor complexity beyond the sialic acid linkage, i.e. binding to a distinct topology that a glycan receptor adopts based on a variety of determinants such as sequence, linkage, chain length, and branching of sugar molecules. Moreover, it has been shown recently that the diversity of sialylated glycans present on RBCs is significantly different from the glycans present in the upper respiratory tract of humans [22]. Other methods such as fetuin capture assays suffer from the same limitation [23].

The continuous progress of chemoenzymatic synthesis strategies for glycans and development of glycan array platforms has enabled the study of HA specificity using chemically defined glycans [24-27]. Intact viruses, recombinantly expressed hemagglutinins, and their mutant forms from H1, H3, and H5 subtypes have already been analyzed using glycan arrays [26,28]. While high-quality binding data can be obtained using such arrays, they do not readily lend themselves as a routine tool for virus surveillance due to three major factors: first, the microarrays are synthesized by molecular printing on glass slides using high-precision equipment, and are still costly to manufacture; second, the glycans are covalently bound to the glass, making the array irreversibly rigid and thus not suitable for rapid construction of a custom-made array; and third, typical array formats are interpreted in an on/off manner, rather than through a quantitative readout, thus missing potentially critical information.

Here we present an alternative to the planar glycan array using magnetic polystyrene microspheres as a flowing matrix for a modular glycan array. Suspension bead arrays offer the advantages of higher flexibility, faster reaction kinetics and greater sensitivity owing to the three-dimensional presentation of glycans. Importantly, flow cytometry enables automated, large-scale sample screening. In the past, microspheres in conjunction with flow cytometry have been used for immunoassays, including for the detection of infectious agents such as influenza [29]. Using custom-designed glycospheres, we developed an assay platform for high-throughput functional characterization of clinical influenza isolates based on their ability to bind to certain host-specific glycan motifs.

## Results and discussion

### Glycan motif selection

**Figure 1:**
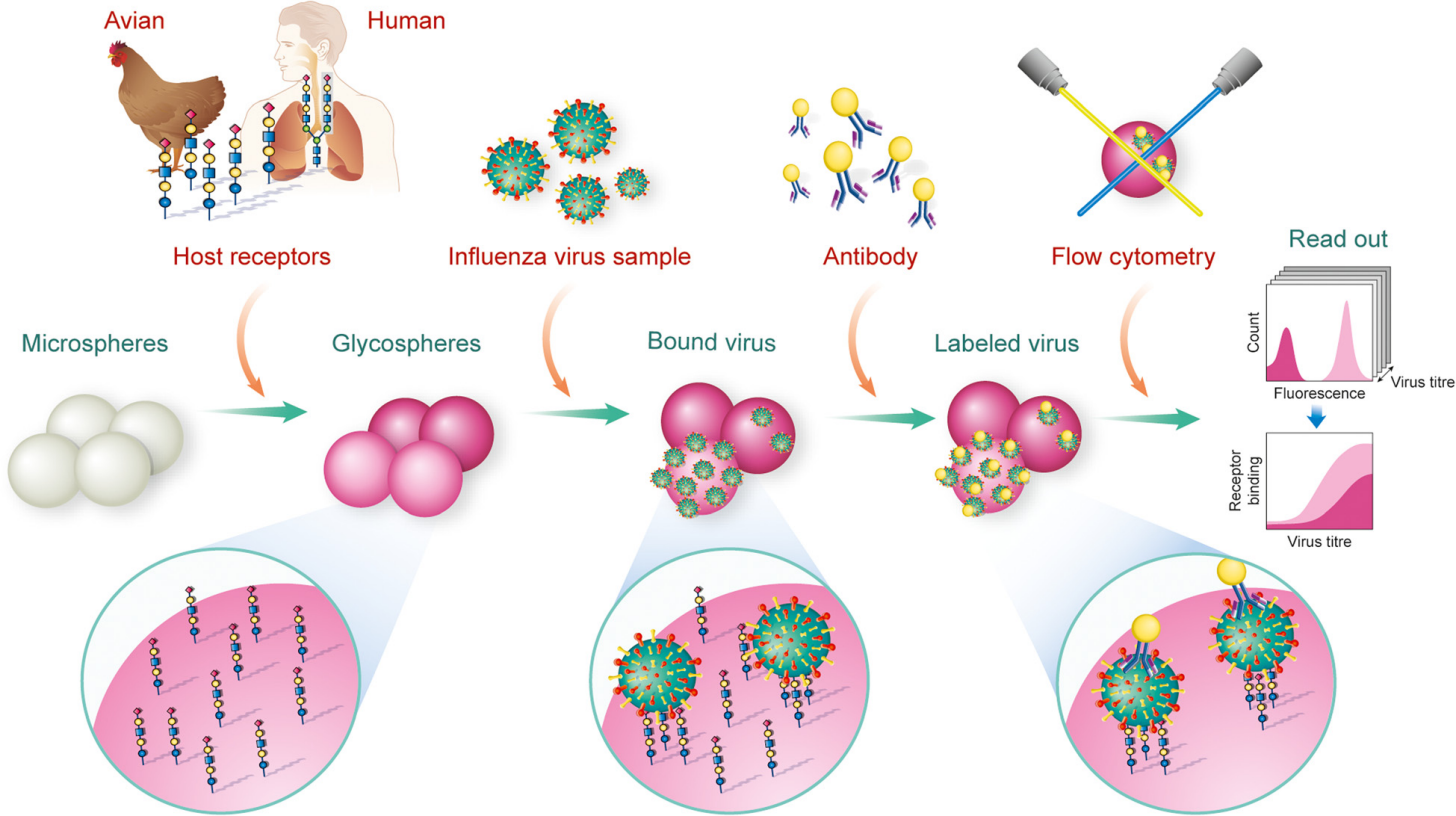
Illustration of an influenza glycosphere assay.

We have previously reported the glycan diversity in human upper respiratory tissue with a predominance of α2-6 sialylated glycans [11]. Further, analysis of glycan array data of human-adapted influenza viruses revealed them to consistently bind to long α2-6 sialylated glycans (tetrasaccharide or longer), despite showing a remarkable overall heterogeneity in their glycan binding patterns, [28,30-36] (summarized in Additional file 2). Together, based on these findings, a commonly available α2-6-sialylated tetrasaccharide glycan, LS-tetrasaccharide c (LSTc), was chosen as a representative influenza receptor of the human upper respiratory tissue. Likewise, a structurally related α2-3 sialylated glycan, LS-tetrasaccharide a (LSTa), was chosen as representative receptor for the binding preference of avian influenza viruses [28,37] (Additional file 2). LSTc and LSTa were biotinylated with a long-chain spacer and purified by HPLC to remove excess biotin. Conjugation of streptavidin-coated microspheres with either of the biotinylated receptors resulted in receptor-specific glycospheres that were used to capture recombinant hemagglutinin or virus from a small sample volume. Bound analyte was then labelled with either a broad-spectrum or subtype-specific antibody and quantified by fluorescent flow cytometry. Figure 1 illustrates the overall assay schematic.

The glycan density on the microspheres (controlled by streptavidin density on the microsphere) was found to be a critical parameter for sensitivity of hemagglutinin binding. The receptor binding domain of a single HA monomer binds to sialylated glycans with very weak affinity, and strong avidity to host cell receptors is only achieved by polyvalent binding of multiple HA trimers [38,39]. Several commercially available preparations of streptavidin-conjugated microspheres were tested and the microspheres with a high streptavidin density provided the highest sensitivity for detection of lectin and virus binding (Additional file 3).

### Functional assessment of glycosphere receptors

**Figure 2:**
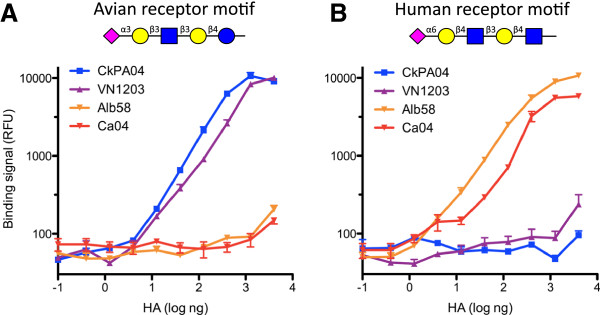
**Binding specificity of the representative receptor motifs used in the glycosphere assay. A**, Recombinantly expressed hemagglutinin of the avian-adapted influenza strains A/Chicken/PA/2004 (CkPA04, H2N2) and A/Vietnam/1203/2004 (Viet04, H5N1) binds quantitatively and selectively to its cognate avian glycan domain. **B**, Hemagglutinin of the human pandemic strains A/Albany/6/58 (Alb58, H2N2) and A/California/04/2009 (Ca04, H1N1) binds with high specificity to only its cognate human glycan domain.

Previous studies have reported that glycans adopt a distinct topology in the presence of HA [11,28,40,41]. The topology is a function of the linkage of the terminal sialic acid with the penultimate monosaccharide, the length of the oligosaccharide, and its binding mode with HA. High-affinity binding to oligosaccharides with α2-6-linked sialic acid that are tetrasaccharide or longer (such as LSTc) has been found to be a key feature for human adaption and human-to-human aerosol transmission [11]. To provide proof of concept of our glycosphere assay, we tested the binding characteristics of two human-adapted and two avian-adapted hemagglutinins whose glycan-binding characteristics have been studied in detail. LSTc and LSTa were used to probe the glycan specificity of human-adapted HAs from the Asian H2N2 pandemic of 1957–58 (A/Albany/6/1958; Alb58) and the H1N1 “swine flu” pandemic of 2009 (A/California/04/2009; Ca04). In addition we tested avian-adapted HAs of the H5N1 bird flu and an avian H2N2 strain from 2004 (A/Vietnam/1203/04, VN1203; and A/Chicken/PA/2004, CkPA04, respectively). Glycospheres with the avian-receptor motif LSTa bound HAs from both VN1203 and CkPA04 in a dose-dependent fashion, with little binding of HAs from Alb58 and Ca04 to the avian receptor (Figure 2A). Conversely, HAs from human viruses Alb58 and Ca04 bound with high affinity to LSTc-glycospheres with little or no binding of HAs from VN1203 and CkPA04 to the human receptor (Figure 2B). These results are consistent with previous findings on planar glycan arrays, and thus served to validate the glycosphere assay as a reliable tool for the functional characterization of influenza binding to host cell receptors.

### Virus characterization

**Figure 3:**
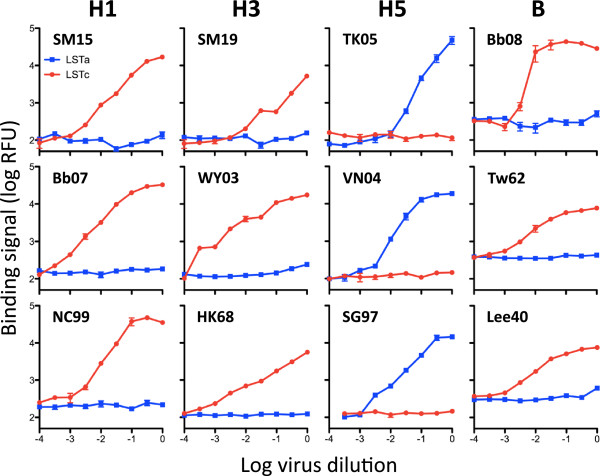
**Quantitative receptor binding analysis of a representative panel of influenza viruses.** Influenza A subtypes H1N1, H3N2, as well as influenza B showed a selective and dose-dependent binding to glycospheres coated with the human receptor motif LSTc (red). H5 strains of both human and avian origin demonstrated highly selective binding to the avian receptor motif LSTa (blue). SM15, A/Singapore/SM15/09; Bb07, A/Brisbane/59/07; NC99, A/New Caledonia/20/99; SM19, A/Singapore/SM19/09; WY03, A/Wyoming/03/03; HK68, A/Hong Kong/8/68; TK05, A/turkey/Turkey/1/05; VN04, A/Vietnam/1194/04; SG97, A/duck/Singapore/97; Bb08, B/Brisbane/60/08; Tw62, B/Taiwan/2/62; Lee40, B/Lee/40. Full details of influenza strains used in this study are provided in Additional file 4.

While quantitative assessment of the HA-glycan interaction for known influenza strains is an important validation of the assay platform, the glycosphere assays are designed to probe the glycan specificity of clinical or veterinarian influenza isolates. A panel of representative influenza A viruses was applied to the glycospheres for receptor-specific binding and incubated with clade/subtype-specific antibodies targeting a conserved region on the stem of HA. Captured virus-antibody complexes were probed with a phycoerythrin-labeled secondary antibody and the glycosphere suspensions were then analyzed by quantitative flow cytometry (Figure 1). Glycosphere analysis revealed specific binding of the H1N1 and H3N2 strains to the human receptor motif LSTc (Figure 3), which is in agreement with the fact that these strains were originally isolated from human patient samples. Importantly, the glycosphere assay worked equally well with live and inactivated virus as it did with purified hemagglutinin alone (Additional file 4). The functional characterization of a clinical isolate of the 2009 H1N1 pandemic (SM15) revealed a binding specificity and relative affinity (Figure 3) that is consistent with the binding of Ca04 hemagglutinin (Figure 2). The H5N1 strain A/Vietnam/1194/04(H5N1) (VN1194) was found to bind to the avian but not the human receptor motif with a binding pattern (Figure 3) identical to the related hemagglutinin of VN1203 (Figure 2).

### Assay performance characteristics

**Figure 4:**
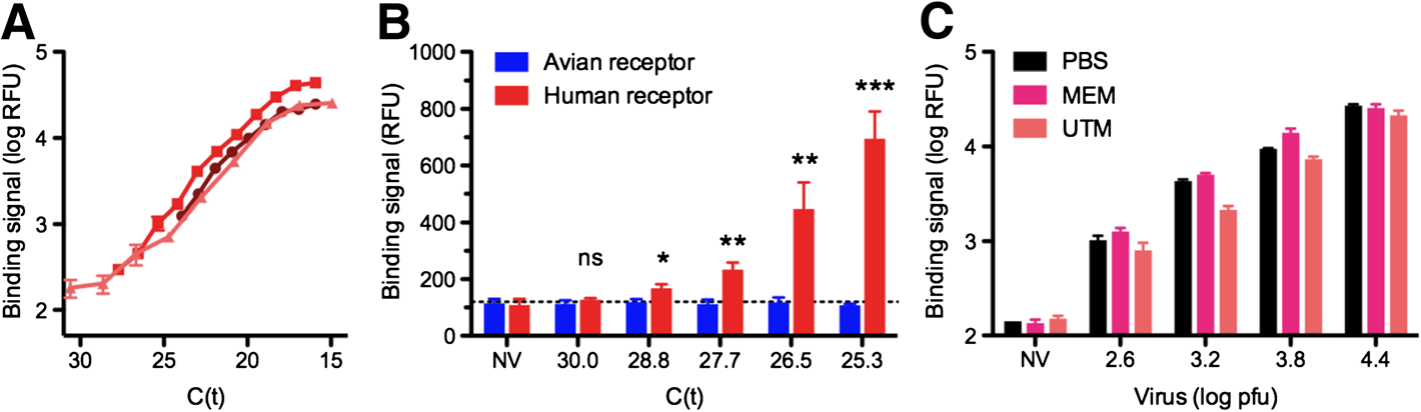
**Assay performance characteristics. A**, Assay reproducibility as determined by three independent receptor binding assays performed in different weeks with independently prepared reagents and non-identical virus stocks of A/Fort Monmouth/1/1947(H1N1). **B**, Limit of detection as determined by comparing the fluorescent signals of increasing viral titers of A/Hong Kong/8/1968(H3N2) with the no-virus control (NV). Statistical significance was determined by an unpaired t-test and categorized as not significant (ns), p < 0.05 (*), p < 0.01 (**), or p < 0.001 (***). **C**, Assay robustness as determined by comparing the signal intensity of identical viral titers in different assay media. A/Fort Monmouth/1/1947(H1N1) was well detected over a range of viral titers when serially diluted in phosphate buffered saline (PBS), virus growth medium (MEM), or Universal Transport Medium (UTM).

To determine the most important assay performance characteristics of the glycosphere assay platform, we studied the reproducibility, limit of detection, and robustness of glycosphere assays. Repeating the assay for selected viral isolates at different times, with independently prepared reagents and non-identical virus stocks, revealed the reliability of the assay platform and the reproducibility of receptor-binding characteristics (Figure 4A).

To determine the limit of detection of the glycosphere assay, we prepared a serial dilution of influenza A virus to run low viral titers in the glycosphere array. Since clinical influenza isolates are routinely quantified by qRT-PCR, we wanted to specify the limit of detection in units of threshold cycle (C_t_) determined by standard diagnostic procedures used in the clinic [42,43]. The limit of detection was consistently in the range of C_t_ values between 25 and 29 (Figure 4B). The relation of C_t_ values, plaque-forming units, and TCID_50_ is provided in Additional file 4.

Further, the glycosphere assay was probed for its robustness towards various sample media. Both virus growth medium and Universal Transport Medium (UTM), which is used in clinical laboratories to collect nasopharyngeal and throat swabs, did not significantly interfere with assay sensitivity and the results were comparable to virus in PBS (Figure 4C).

## Conclusions

We introduce here a high-throughput suspension array platform using glycan-coated microspheres for the functional characterization of clinical and veterinarian influenza isolates. The use of two distinct influenza receptor glycans enabled us to rapidly determine the binding preference of a virus and thus assess the level of adaptation of the virus hemagglutinin to human host cells. We anticipate that the assay developed here will complement traditional genotyping assays and provide a functional assessment of the viruses for better surveillance of emerging influenza strains.

## Methods

### Biotinylation of glycans

LS-tetrasaccharide c (LSTc) and LS-tetrasaccharide a (LSTa; Isosep AB) were biotinylated with EZ-Link Biotin-LC-Hydrazide (Thermo) according to the manufacturer’s instructions. Biotinylated glycans were collected on a GlykoClean G Cartridge (Prozyme), eluted with water, lyophilized, and further purified by separating the biotinylated glycans from free biotin by HPLC. HPLC separation was done with a GLYCOSEP™ N HPLC column (Prozyme), running an increasing gradient (20-53%) of 50 mM ammonium formate pH 4.4 in acetonitrile. The collected glycan fraction was lyophilized, reconstituted twice in water to remove residual salts, and analyzed for purity and integrity by MALDI-TOF (Additional file 5). Purified biotinylated glycans in solution were quantified by both a Sialic Acid Quantification Kit (Prozyme) and a HABA Biotin Quantitation Kit (AnaSpec), following the manufacturers’ instructions. The biotin and sialic acid concentrations differed less than 1% from each other.

### Preparation and analysis of glycospheres

Glycospheres were prepared by incubating streptavidin-functionalized polystyrene beads with biotinylated glycans for one hour at room temperature. The glycosylated beads were washed twice with assay buffer (PBS-1% BSA) to remove excess glycans. The glycospheres were then incubated with recombinant hemagglutinin, live virus, or inactivated virus as described in detail below. After analyte binding, the glycospheres were washed twice each with wash buffer (PBS 0.1% Tween) and assay buffer. Glycospheres were then analyzed on a BD LSRII flow cytometer with blue (488 nm, 100 mW, LP505, BP525/50) and yellow-green (561 nm, 50 mW, LP570, BP585/15) laser. A comparison of different streptavidin microspheres revealed a remarkable range of biotin-binding capacities (Additional file 3). Singlets of high-capacity paramagnetic glycospheres were gated from duplets and multiplets by forward and side scatter (Additional file 6), and the mean signal intensity of microsphere singlets was further analyzed. Non-glycosylated microspheres were used as a negative control to assess non-specific binding of analyte. Every binding assay was performed over a range of analyte concentration and each concentration was tested in at least three independent assay reactions. The absolute and relative binding signal intensity (mean ± standard deviation, n ≥ 3) was plotted against analyte concentration. Concentration-dependent binding was verified by a linear response when plotting on double-logarithmic scale. GraphPad Prism 5.0 was used for data plotting and statistical analyses.

### Hemagglutinin and influenza virus

Soluble hemagglutinin was recombinantly expressed in a baculovirus system as described previously [11]. Details of viruses used in this study are listed in Additional file 4. Clinical isolates A/Singapore/SM15/2009(H1N1) and A/Singapore/SM19/2009(H3N2) were a kind gift of Julian Tang, Evelyn Koay, and Paul Tambyah from the National University Health System (NUHS), Singapore. Influenza viruses A/New Caledonia/20/99(H1N1), A/Vietnam/1194/2004(H5N1), and A/duck/Singapore/97(H5N3) were purchased as inactivated virus (Fitzgerald Inc.; NIBSC). All other viruses were obtained from the American Type Culture Collection (ATCC). Virus propagation in MDCK cells, harvesting, and titer determination by both plaque and TCID_50_ assays were performed using established standard procedures [44], with the exception of using Avicel RC-591F (FMC BioPolymer) instead of agarose overlay in the plaque assays [45]. Viral RNA was quantified by qRT-PCR according to the protocol by Spackman et al. [43], with slight adaptations in primer sequence for quantification of 2009 H1N1 strains [42].

### Antibody selection

Antibodies for the quantitative detection and labelling of captured virus were selected based on their ability to bind to a conserved region on a wide variety of strains within a subtype or clade. Further, the antibodies were chosen such that the antibody binding does not interfere with receptor binding function of the hemagglutinin on the virus. The modular design of the glycosphere assay allows for a choice of subtype specific, clade specific, or universal HA antibodies that target a conserved region on the stem of HA. In the present study, mouse monoclonal antibody clones C179 and F49 (Takara) were used as clade 1 (H1N1, H2N2, H5N1) and clade 2 (H3N2) specific antibodies, respectively. A rabbit polyclonal antibody against human influenza A and B (Takara) was used to detect influenza B virus. Goat anti-mouse secondary antibody conjugated to R-phycoerythrin (Invitrogen) was used as fluorescent marker for flow cytometry analysis.

### Hemagglutinin binding

To account for the avidity in hemagglutinin binding to sialylated glycans, recombinant hemagglutinin was pre-complexed with primary and secondary antibody in a 4:2:1 ratio as described previously [16]. In brief, hemagglutinin, mouse anti-6X His IgG (Abcam), and RPE-conjugated goat anti-mouse Ab were mixed in a ratio of 4:2:1 and kept on ice for 20 min. The resulting precomplex was then topped up with assay buffer to a final HA concentration of 4 μg of HA per assay. A half-log serial dilution of the precomplex in assay buffer was prepared and applied to the glycospheres. After gently rotating the beads for two hours at room temperature, the beads were washed and analyzed as described above.

### Virus binding

Undiluted culture supernatant was used in the glycosphere assays to determine glycan binding at the highest viral titer available for each strain. Twofold or halflog serial dilutions of virus in assay buffer were incubated with 1 pmol primary antibody per assay for 1 hour at room temperature. Virus-antibody aggregates were mixed thoroughly and applied to the glycospheres on ice. After overnight incubation at 4°C, the glycospheres were washed twice each with cold wash and assay buffer, and RPE-conjugated goat anti-mouse IgG (Invitrogen) was added at 0.2 μg per assay. Following incubation at 4°C for another 2 hours, the glycospheres were washed and analyzed as described above.

## Competing interests

The authors declare that they have no competing interests.

## Authors’ contributions

SH, KV, IB, ZS and RS wrote the manuscript. SH, KV, IB, UA, VS and RS contributed reagents/materials/analysis tools. SH, KV, IB, ZS, RR and RS analyzed the data. SH, IB and UA performed the experiments. SH, KV, IB, UA, ZS, VS, RR and RS conceived and designed the experiments. All authors read and approved the final manuscript.

## Supplementary Material

Additional file 1: Table S1 Correlation of receptor binding avidities with virus transmissibility. Click here for file

Additional file 2: Table S2 Glycan array affinities of influenza virus to human and avian receptor glycans. Click here for file

Additional file 3: Figure S1 Comparison of different streptravidin-conjugated microspheres with regards to their biotin-binding capacities and performance in glycosphere assays. Click here for file

Additional file 4: Table S3 Influenza viruses used in this study. Click here for file

Additional file 5: Figure S2 MALDI-MS spectra of purified LST-LC-biotin. Click here for file

Additional file 6: Figure S3 Gating of paramagnetic microspheres during flow cytometry analysis. Click here for file
