# Supplementary material for: Modular glycosphere assays for high-throughput functional characterization of influenza viruses
Source: BMC Biotechnol. 2013 Apr 15;13:34. doi: 10.1186/1472-6750-13-34 (PMC3751502; doi:10.1186/1472-6750-13-34)
Supplement: Additional file 1: Table S1 — Correlation of receptor binding avidities with virus transmissibility. [file 1472-6750-13-34-S1.pdf]

**Additional file 1. Table S1: Correlation of receptor binding avidities with virus transmissibility**

| Virus strain | Affinity to glycan topology: Cone ($\alpha$2,3 and short $\alpha$2,6) | Umbrella (long $\alpha$2,6) | $K_d'$ | Transmissibility | Reference |
|---|---|---|---|---|---|
| H1N1 | | | | | |
| Spanish flu (SC18) | – | +++ | 5.5 pM | +++ | [1] |
| Spanish flu (NY18) | + | ++ | 6.3 nM | ++ | [1] |
| 2009 H1N1 (‘Swine flu’) | – | ++ | 1.5 nM | ++ | [2] |
| H2N2 | | | | | |
| Asian flu (Alb58) | ++ | +++ | 5.1 pM | +++ | [3, 4] |
| Asian flu (ElSal57) | +++ | – | low | – | [3, 4] |
| H5N1 | | | | | |
| Bird flu (Viet04) | +++ | – | low | – | [5] |
| Bird flu (HK486) | +++ | – | low | – | [5] |

## References


1. Srinivasan A, Viswanathan K, Raman R, Chandrasekaran A, Raguram S, Tumpey TM, Sasisekharan V, Sasisekharan R: **Quantitative biochemical rationale for differences in transmissibility of 1918 pandemic influenza A viruses.** *Proc Natl Acad Sci U S A* 2008, **105:**2800-2805.
2. Maines TR, Jayaraman A, Belser JA, Wadford DA, Pappas C, Zeng H, Gustin KM, Pearce MB, Viswanathan K, Shriver ZH, et al: **Transmission and pathogenesis of swine-origin 2009 A(H1N1) influenza viruses in ferrets and mice.** *Science* 2009, **325:**484-487.
3. Pappas C, Viswanathan K, Chandrasekaran A, Raman R, Katz JM, Sasisekharan R, Tumpey TM: **Receptor specificity and transmission of H2N2 subtype viruses isolated from the pandemic of 1957.** *PLoS One* 2010, **5:**e11158.
4. Viswanathan K, Koh X, Chandrasekaran A, Pappas C, Raman R, Srinivasan A, Shriver Z, Tumpey TM, Sasisekharan R: **Determinants of glycan receptor specificity of H2N2 influenza A virus hemagglutinin.** *PLoS One* 2010, **5:**e13768.
5. Chandrasekaran A, Srinivasan A, Raman R, Viswanathan K, Raguram S, Tumpey TM, Sasisekharan V, Sasisekharan R: **Glycan topology determines human adaptation of avian H5N1 virus hemagglutinin.** *Nat Biotechnol* 2008, **26:**107-113.
