# Supplementary material for: Modular glycosphere assays for high-throughput functional characterization of influenza viruses
Source: BMC Biotechnol. 2013 Apr 15;13:34. doi: 10.1186/1472-6750-13-34 (PMC3751502; doi:10.1186/1472-6750-13-34)
Supplement: Additional file 2: Table S2 — Glycan array affinities of influenza virus to human and avian receptor glycans. [file 1472-6750-13-34-S2.pdf]

Additional file 2. Table S2: Affinity of influenza virus to human and avian receptor motifs in glycan arrays

Influenza virus	Binding affinity to glycans that terminate in		CFG primary screen no./Reference
	Neu5Acα2-6Galβ1-4GlcNAcβ1-3Gal (LSTc-like) 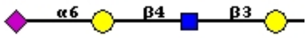	Neu5Acα2-3Galβ1-3/4GlcNAc (LSTa-like) 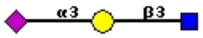	
**Human H1N1**			
A/Oklahoma/1707/2010(H1N1)	High	ns	PS_5253
A/California/04/2009(H1N1)	High	ns	[1]
A/Brisbane/59/2007(H1N1)	High	Low	[1]
A/New Caledonia/20/1999	High	Low	[1]
A/Texas/36/1991(H1N1)	High	Low	[2, 3]
A/New Jersey/8/1976(H1N1)	High	High	PS_3815
A/Puerto Rico/8/1934(H1N1)	High	High	PS_1367
A/South Carolina/1/1918 (H1N1)	High	ns	[2, 3]
**Human H3N2**			
A/Oklahoma/5342/2010(H3N2)	High	High	PS_5231
A/Oklahoma/483/2008(H3N2)	High	ns	[4]
A/Uruguay/716/2007 (H3N2)	High	High	PS_5251
A/Oklahoma/309/2006(H3N2)	High	High	[4]
A/New York/55/2004(H3N2)	High	ns	[5]
A/Wyoming/3/2003(H3N2)	High	Low	[6]
A/Moscow/10/1999(H3N2)	High	ns	[2, 3]
A/Philippines/2/1982 (H3N2)	High	High	[6]
A/Aichi/2/1968(H3N2)	High	Low	[7]
**Avian Influenza A**			
A/Chicken/PA/2004(H2N2)	ns	High	PS_3482
A/Duck/Singapore/3/1997(H5N3)	ns	High	[8]
A/Turkey/MO/21939/1987(H1N1)	ns	High	PS_3478
A/Duck/Hong Kong/562/1979(H10N9)	ns	High	PS_3828
A/Duck/Alberta/35/1976(H1N1)	ns	High	[8]
A/Duck/Ukraine/1/1963(H3N8)	ns	High	[2]
**Human influenza B**			
B/Michigan/2006	High	Low	PS_789
B/Victoria/504/2000	High	Low	[9]
B/Maryland/1/1959	High	ns	[10]

CFG, Consortium for Functional Glycomics, http://www.functionalglycomics.org
ns, not significant

References

1. Bradley KC, Jones CA, Tompkins SM, Tripp RA, Russell RJ, Gramer MR, Heimburg-Molinaro J, Smith DF, Cummings RD, Steinhauer DA: **Comparison of the receptor binding properties of contemporary swine isolates and early human pandemic H1N1 isolates (Novel 2009 H1N1).** *Virology* 2011, **413:**169-182.
2. Stevens J, Blixt O, Glaser L, Taubenberger JK, Palese P, Paulson JC, Wilson IA: **Glycan microarray analysis of the hemagglutinins from modern and pandemic influenza viruses reveals different receptor specificities.** *J Mol Biol* 2006, **355:**1143-1155.
3. Chandrasekaran A, Srinivasan A, Raman R, Viswanathan K, Raguram S, Tumpey TM, Sasisekharan V, Sasisekharan R: **Glycan topology determines human adaptation of avian H5N1 virus hemagglutinin.** *Nat Biotechnol* 2008, **26:**107-113.
4. Gulati S, Smith DF, Air GM: **Deletions of neuraminidase and resistance to oseltamivir may be a consequence of restricted receptor specificity in recent H3N2 influenza viruses.** *Virol J* 2009, **6:**22.
5. Oshansky CM, Pickens JA, Bradley KC, Jones LP, Saavedra-Ebner GM, Barber JP, Crabtree JM, Steinhauer DA, Tompkins SM, Tripp RA: **Avian influenza viruses infect primary human bronchial epithelial cells unconstrained by sialic acid alpha2,3 residues.** *PLoS One* 2011, **6:**e21183.
6. Kumari K, Gulati S, Smith DF, Gulati U, Cummings RD, Air GM: **Receptor binding specificity of recent human H3N2 influenza viruses.** *Virol J* 2007, **4:**42.
7. Bradley KC, Galloway SE, Lasanajak Y, Song X, Heimburg-Molinaro J, Yu H, Chen X, Talekar GR, Smith DF, Cummings RD, Steinhauer DA: **Analysis of influenza virus hemagglutinin receptor binding mutants with limited receptor recognition properties and conditional replication characteristics.** *J Virol* 2011, **85:**12387-12398.
8. Stevens J, Blixt O, Tumpey TM, Taubenberger JK, Paulson JC, Wilson IA: **Structure and receptor specificity of the hemagglutinin from an H5N1 influenza virus.** *Science* 2006, **312:**404-410.
9. Lugovtsev VY, Smith DF, Weir JP: **Changes of the receptor-binding properties of influenza B virus B/Victoria/504/2000 during adaptation in chicken eggs.** *Virology* 2009, **394:**218-226.
10. Triana-Baltzer GB, Sanders RL, Hedlund M, Jensen KA, Aschenbrenner LM, Larson JL, Fang F: **Phenotypic and genotypic characterization of influenza virus mutants selected with the sialidase fusion protein DAS181.** *J Antimicrob Chemother* 2011, **66:**15-28.
