# Supplementary material for: Modular glycosphere assays for high-throughput functional characterization of influenza viruses
Source: BMC Biotechnol. 2013 Apr 15;13:34. doi: 10.1186/1472-6750-13-34 (PMC3751502; doi:10.1186/1472-6750-13-34)
Supplement: Additional file 3: Figure S1 — Comparison of different streptravidin-conjugated microspheres with regards to their biotin-binding capacities and performance in glycosphere assays. [file 1472-6750-13-34-S3.pdf]

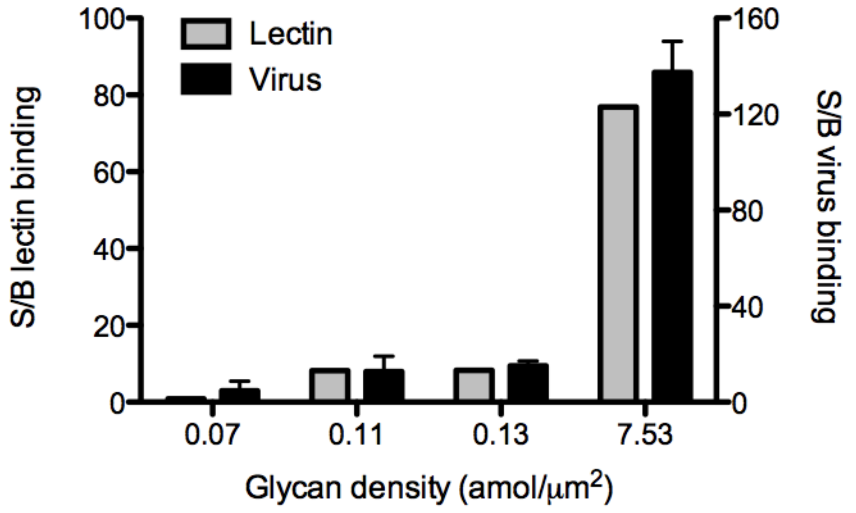

Additional file 3. Figure S1: Comparison of different streptravidin-conjugated microspheres with regards to their biotin-binding capacities and performance in glycosphere assays. The glycan density per surface area was calculated by dividing the measured biotin-binding capacity by the surface area of microspheres. Glycosphere performance in lectin (*Sambucus nigra* bark lectin, SNA) and virus binding assays is provided as signal-over-background (S/B).
