# Supplementary material for: Modular glycosphere assays for high-throughput functional characterization of influenza viruses
Source: BMC Biotechnol. 2013 Apr 15;13:34. doi: 10.1186/1472-6750-13-34 (PMC3751502; doi:10.1186/1472-6750-13-34)
Supplement: Additional file 4: Table S3 — Influenza viruses used in this study. [file 1472-6750-13-34-S4.pdf]

Additional file 4. Table S3: **Influenza viruses used in this study**

				Titre		
**Acronym**	**Strain**	**Subtype**	**Ref./Source**	**C(t)**	**Pfu/mL**	**$TCID_{50}$/mL**
	**Live virus**					
SM15	A/Singapore/SM15/2009	H1N1	This study[a]	13.7	4×10^{7}	*nd*[c]
SM19	A/Singapore/SM19/2009	H3N2	This study[b]	18.0	3×10^{4}	*nd*[c]
FM47	A/Fort Monmouth/1/1947	H1N1	ATCC VR-97	15.9	3×10^{4}	$10^{5.4}$
Ai68	A/Aichi/2/1968	H3N2	ATCC VR-1680	15.0	1×10^{6}	$10^{7.7}$
HK68	A/HongKong/8/1968	H3N2	ATCC VR-1679	19.4	1×10^{5}	$10^{4.7}$
Lee40	B/Lee/1940	B	ATCC VR-1535	23.9	1×10^{9}	*nd*[c]
Tw62	B/Taiwan/2/1962	B	ATCC VR-1735	24.2	1×10^{4}	*nd*[c]
	**Inactivated virus**					
Bb07	A/Brisbane/59/2007	H1N1	NIBSC			
NC99	A/New Caledonia/20/1999	H1N1	Fitzgerald Inc.			
WY03	A/Wyoming/03/2003	H3N2	NIBSC			
TK05	A/turkey/Turkey/1/2005	H5N1	NIBSC			
VN04	A/Vietnam/1194/2004	H5N1	NIBSC			
SG97	A/duck/Singapore/1997	H5N3	NIBSC			
Bb08	B/Brisbane/60/2008	B	NIBSC			
	**Recombinant HA**					
Ca04	A/California/04/2009	H1N1	[1]			
Alb58	A/Albany/1958	H2N2	[2]			
CkPA04	A/chicken/Pennsylvania/2004	H2N2	[3]			
VN1203	A/Vietnam/1203/2004	H5N1	[4]			

[a]Genbank sequence accession number of the HA gene, JX844664

[b]Genbank sequence accession number of the HA gene, JX844665

[c]*nd*, not determined

References

1. Maines TR, Jayaraman A, Belser JA, Wadford DA, Pappas C, Zeng H, Gustin KM, Pearce MB, Viswanathan K, Shriver ZH, et al: **Transmission and pathogenesis of swine-origin 2009 A(H1N1) influenza viruses in ferrets and mice.** *Science* 2009, **325:**484-487.
2. Pappas C, Viswanathan K, Chandrasekaran A, Raman R, Katz JM, Sasisekharan R, Tumpey TM: **Receptor specificity and transmission of H2N2 subtype viruses isolated from the pandemic of 1957.** *PLoS One* 2010, **5:**e11158.
3. Viswanathan K, Koh X, Chandrasekaran A, Pappas C, Raman R, Srinivasan A, Shriver Z, Tumpey TM, Sasisekharan R: **Determinants of glycan receptor specificity of H2N2 influenza A virus hemagglutinin.** *PLoS One* 2010, **5:**e13768.
4. Chandrasekaran A, Srinivasan A, Raman R, Viswanathan K, Raguram S, Tumpey TM, Sasisekharan V, Sasisekharan R: **Glycan topology determines human adaptation of avian H5N1 virus hemagglutinin.** *Nat Biotechnol* 2008, **26:**107-113.
