# Supplementary material for: Modular glycosphere assays for high-throughput functional characterization of influenza viruses
Source: BMC Biotechnol. 2013 Apr 15;13:34. doi: 10.1186/1472-6750-13-34 (PMC3751502; doi:10.1186/1472-6750-13-34)
Supplement: Additional file 5: Figure S2 — MALDI-MS spectra of purified LST-LC-biotin. [file 1472-6750-13-34-S5.pdf]

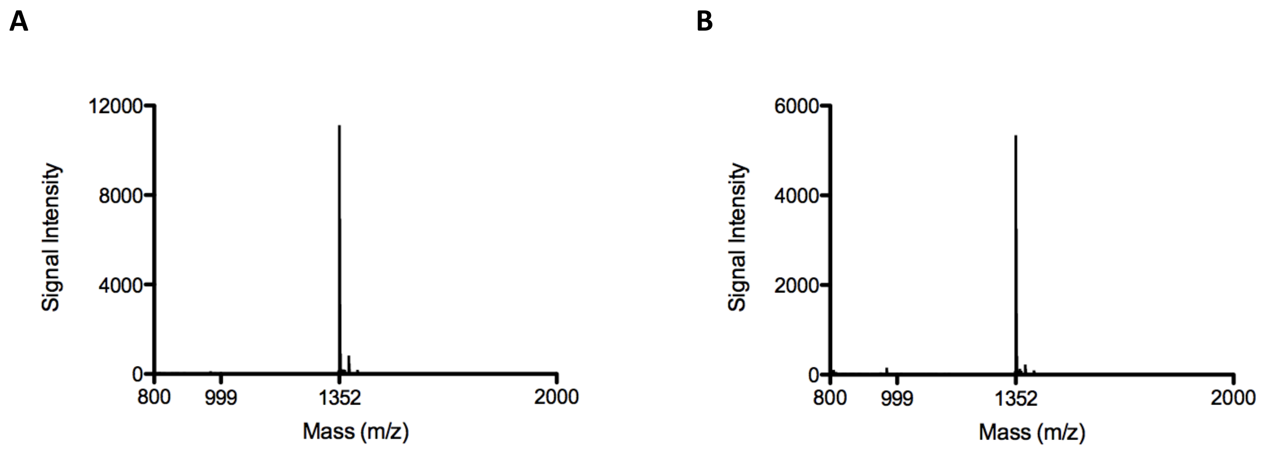


**Additional file 5. Figure S1*:*** MALDI-MS spectra of purified LST-LC-biotin. **A**, LSTc (Neu5Ac(α2-6)Gal(β1-4)GlcNAc(β1-3)Gal(β1-4)Glc-LC-biotin; MW = 1352). **B**, LSTa (Neu5Ac(α2-3)Gal(β1-3)GlcNAc(β1-3)Gal(β1-4)Glc-LC-biotin; MW = 1352). The molecular weight of free LSTc and LSTa is 998.9.
