# Supplementary material for: Modular glycosphere assays for high-throughput functional characterization of influenza viruses
Source: BMC Biotechnol. 2013 Apr 15;13:34. doi: 10.1186/1472-6750-13-34 (PMC3751502; doi:10.1186/1472-6750-13-34)
Supplement: Additional file 6: Figure S3 — Gating of paramagnetic microspheres during flow cytometry analysis. [file 1472-6750-13-34-S6.pdf]

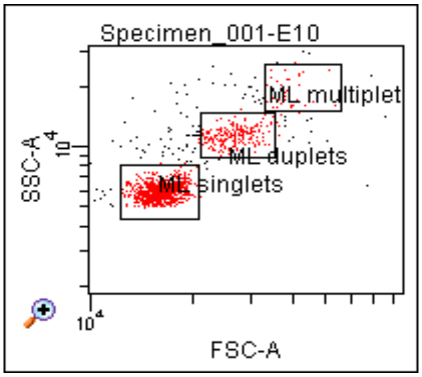

Additional file 6. Figure S3: Gating of paramagnetic microspheres during flow cytometry analysis. Forward and side scatter was used to gate microsphere singlets, duplets from multiplets. Identification of singlets and duplets was confirmed by microscopy and the fact that the average signal intensity per duplet event was twice as high as the average signal intensity per singlet event.
